# Identification and validation of *SERPINE1* as a prognostic and immunological biomarker in pan-cancer and in ccRCC

**DOI:** 10.3389/fphar.2023.1213891

**Published:** 2023-08-23

**Authors:** Lingqin Li, Fan Li, Zhehao Xu, Liyang Li, Haiyi Hu, Yang Li, Shicheng Yu, Mingchao Wang, Lei Gao

**Affiliations:** ^1^ Department of Operating Room, Sir Run Run Shaw Hospital, Zhejiang University School of Medicine, HangZhou, China; ^2^ Department of Urology, Sir Run Run Shaw Hospital, Zhejiang University School of Medicine, Hangzhou, China; ^3^ University of New South Wales, School of Medicine, Sydney, NSW, Australia

**Keywords:** *SERPINE1*, pan-cancer, multi-omics, clear cell renal cell carcinoma, cancer immunity

## Abstract

**Background:** *SERPINE1*, a serine protease inhibitor involved in the regulation of the plasminogen activation system, was recently identified as a cancer-related gene. However, its clinical significance and potential mechanisms in pan-cancer remain obscure.

**Methods:** In pan-cancer multi-omics data from public datasets, including The Cancer Genome Atlas (TCGA) and Genotype-Tissue Expression (GTEx), and online web tools were used to analyze the expression of *SERPINE1* in different cancers and its correlation with prognosis, genetic alteration, DNA promoter methylation, biological processes, immunoregulator expression levels, immune cell infiltration into tumor, tumor mutation burden (TMB), microsatellite instability (MSI), immunotherapy response and drug sensitivity. Further, two single-cell databases, Tumor Immune Single-cell Hub 2 (TISCH2) and CancerSEA, were used to explore the expression and potential roles of *SERPINE1* at a single-cell level. The aberrant expression of *SERPINE1* was further verified in clear cell renal cell carcinoma (ccRCC) through qRT-PCR of clinical patient samples, validation in independent cohorts using The Gene Expression Omnibus (GEO) database, and proteomic validation using the Clinical Proteomic Tumor Analysis Consortium (CPTAC) database.

**Results:** The expression of *SERPINE1* was dysregulated in cancers and enriched in endothelial cells and fibroblasts. Copy number amplification and low DNA promoter methylation could be partly responsible for high *SERPINE1* expression. High *SERPINE1* expression was associated with poor prognosis in 21 cancers. The results of gene set enrichment analysis (GSEA) indicated *SERPINE1* involvement in the immune response and tumor malignancy. *SERPINE1* expression was also associated with the expression of several immunoregulators and immune cell infiltration and could play an immunosuppression role. Besides, *SERPINE1* was found to be related with TMB, MSI, immunotherapy response and sensitivity to several drugs in cancers. Finally, the high expression of *SERPINE1* in ccRCC was verified using qRT-PCR performed on patient samples, six independent GEO cohorts, and proteomic data from the CPTAC database.

**Conclusion:** The findings of the present study revealed that *SERPINE1* exhibits aberrant expression in various types of cancers and is associated with cancer immunity and tumor malignancy, providing novel insights for individualized cancer treatment.

## 1 Introduction

As one of the leading causes of death worldwide, cancer imposes an immense burden on the human society every year (Sung et al., 2021; Siegel et al., 2023). Despite significant advances in cancer treatment and early screening over the recent years, the overall survival prognosis for patients with cancer remains unsatisfactory, especially in certain cancer types (Sung et al., 2021; Hu et al., 2022). As such, there is an urgent need to explore new targets for cancer diagnosis and treatment.

Serine protease inhibitor clade E member 1 (*SERPINE1*, also known as PAI-1) is a serine protease inhibitor that plays key roles in regulating the plasminogen activation system (Placencio and DeClerck, 2015). By binding to and inactivating tissue-type plasminogen activator (tPA) and urokinase-type plasminogen activator (uPA), *SERPINE1* exerts antifibrinolytic effects (Declerck and Gils, 2013). The inhibition of PAI-1 leads to increased thrombolysis in artery disease (Kohler and Grant, 2000). *SERPINE1* consists of 379 amino acids and is synthesized and secreted primarily by vascular endothelial cells, adipocytes, and platelets (Chen et al., 2021).

In addition to regulating the plasminogen/plasminase system, *SERPINE1* has been found to be involved in a variety of other processes, such as pericellular proteolysis, tissue remodeling, cell migration, inflammation, angiogenesis, and apoptosis, implying its involvement in various diseases (Placencio and DeClerck, 2015; Sillen and Declerck, 2021). In recent years, the abnormal expression of *SERPINE1* has been found in various cancer types. Specifically, *SERPINE1* overexpression has been observed in breast cancer (Duffy et al., 2014; Jevrić et al., 2019), melanoma (Hanekom et al., 2002), non-small cell lung cancer (Sotiropoulos et al., 2019), bladder cancer (Becker et al., 2010), and ovarian cancer (Nakatsuka et al., 2017). The inhibition of *SERPINE1* expression has been shown to impede tumor progression and angiogenesis in several cancer types (Gomes-Giacoia et al., 2013; Masuda et al., 2013; Mashiko et al., 2015; Placencio et al., 2015; Takayama et al., 2016). *SERPINE1*-deficient mice also exhibited delayed tumor development, cancer invasion, and vascularization (Bajou et al., 1998; Gutierrez et al., 2000). Therefore, *SERPINE1* is expected to be a promising novel target for the diagnosis and treatment of cancers. However, the detailed mechanisms underlying the involvement of *SERPINE1* in cancers remain unclear.

Currently, there is no comprehensive study on the role of *SERPINE1* in pan-cancer. In the present study, we performed a multi-omics analysis of *SERPINE1* in pan-cancer. Our results confirmed the aberrant expression of *SERPINE1* in multiple cancers and the relationship of *SERPINE1* expression with the tumor microenvironment and cancer immunity. Furthermore, qRT-PCR was performed to validate the abnormal expression of *SERPINE1* in clear cell renal cell carcinoma samples.

## 2 Materials and methods

### 2.1 The workflow of the study

**FIGURE 1:**
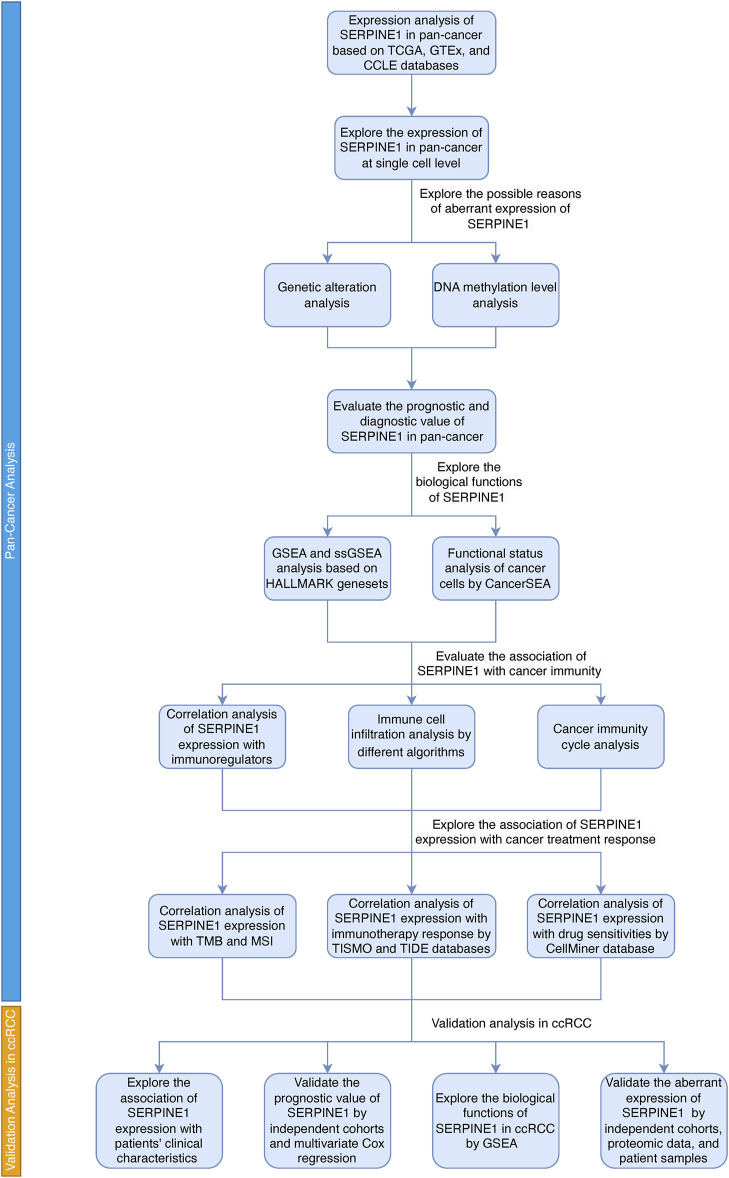
The flow chart of this study.

Based on several public datasets, we analyzed the expression of *SERPINE1* in pan-cancer and explored the possible causes of its abnormal expression. Then we evaluated the prognostic and diagnostic value of *SERPINE1* in pan-cancer to assess its clinical value. Next, we explored the biological functions associated with *SERPINE1* expression and found that it was associated with tumor malignancy and cancer immunity. Co-expression analysis, immune cell infiltration analysis, and cancer immunity cycle analysis were performed as well to validate its association with cancer immunity. Then we analyzed the correlation of *SERPINE1* expression with response of cancer treatments to further explore its clinical value. At last, validation analyses were performed in ccRCC. A summary of the workflow of this study is shown in Figure 1.

### 2.2 Tissue samples and sample size calculation

The Ethics Committee of the Sir Run Run Shaw Hospital, Zhejiang University approved this study, and written informed consent was obtained from all participating patients. The research procedures adhered to the guidelines of the Declaration of Helsinki. For assessing *SERPINE1* expression, 26 tissue samples (13 tumor tissues and 13 paired adjacent normal tissues) were randomly selected from clear cell renal cell carcinoma patients. The power analysis was performed using G*Power (G*Power, version 3.1 for MAC, Dusseldorf, North Rhine-Westphalia). According to the TCGA-KIRC project, the *SERPINE1* expression of tumor samples (with available paired adjacent normal tissue data) were 5.8746 ± 1.6083 (log2FPKM) and the expression of adjacent normal tissues were 3.9290 ± 1.7263 (log2FPKM), indicating a effect size of 0.8615 (Cancer Genome Atlas Research Network, 2013). With power (1-β) of 0.85 and α error of 0.05, the sample size was calculated to be 13 pairs of samples.

### 2.3 Gene expression analysis of *SERPINE1* in pan-cancer

Transcriptional data of tumor and normal samples were collected from the UCSC Xena (https://xenabrowser.net/datapages/) dataset (Goldman et al., 2020). Expression data of cancer cell lines were downloaded from the Cancer Cell Line Encyclopedia (CCLE, https://sites.broadinstitute.org/ccle) (Barretina et al., 2012). The abbreviations of cancer names are presented in Supplementary Table S1. Wilcox rank sum test was used to compare gene expression levels. R (version 4.2.3) and R package “ggplot2” were used for statistical analysis and visualization.

### 2.4 Single-cell analysis of *SERPINE1* expression

Expression of *SERPINE1* at a single-cell level was analyzed using Tumor Immune Single-cell Hub 2 (TISCH2), based on the MAESTRO workflow (Han et al., 2022). The following parameters were used for analysis: *SERPINE1* (Gene), major lineages (Cell-type annotation), and all cancers (Cancer type). The R Package “ComplexHeatmap” (version 2.14.0) was used for data visualization (Gu et al., 2016).

### 2.5 Genetic alteration and DNA methylation analysis

Genetic alteration and DNA methylation data from the TCGA database were download from The cBioPortal (http://cbioportal.org) and GSCA (http://bioinfo.life.hust.edu.cn/GSCA/#/) (Cerami et al., 2012; Liu et al., 2023). Spearman’s rank correlation coefficient was used to evaluate the correlations between copy number variation, the DNA methylation level, and *SERPINE1* expression.

### 2.6 Clinical and prognostic value analysis of *SERPINE1*

Clinical data and different types of prognostic data were extracted from UCSC Xena (https://xenabrowser.net/datapages/) (Goldman et al., 2020). Both Cox regression and Kaplan–Meier analysis were conducted to assess the correlation between *SERPINE1* expression and patient prognosis. The cutoff value of the Kaplan–Meier estimator was determined using the “surv-cutpoint” function of the R package “survminer” (version 0.4.9). Information regarding immune subtypes was obtained from a previous report (Thorsson et al., 2018). Moreover, six independent datasets from BEST (https://rookieutopia.com/) were analyzed to validate the prognostic value of *SERPINE1* expression in clear cell renal cell carcinoma, including the GSE167573, GSE29609, and GSE22541 cohorts from GEO (https://www.ncbi.nlm.nih.gov/), the E-MTAB-1980 cohort from EMBL’s European Bioinformatics Institute (EMBL-EBI, https://www.ebi.ac.uk/), and Renal Cell Cancer-European Union (RECA-EU) project data from the International Cancer Genome Consortium (ICGC, https://dcc.icgc.org/). The diagnostic value of *SERPINE1* was assessed by receiver operating characteristic curve (ROC) using R package “pROC” (version 1.18.4).

### 2.7 Functional enrichment analysis and gene set enrichment analysis of *SERPINE1*

The associations between *SERPINE1* expression and several biological processes were examined using GSEA and ssGSEA. Gene sets were downloaded from the Molecular Signatures Database (MSigDB, https://www.gsea-msigdb.org/gsea/index.jsp). Samples were grouped according to the median *SERPINE1* expression for each cancer in GSEA. The R package “GSVA” (version 1.46.0) was used for the GSEA and ssGSEA analyses.

### 2.8 Single cell-level analysis of *SERPINE1*

CancerSEA, which is based on Gene Set Variance Analysis (GSVA), was used to assess the functional status of cancer cells at the single-cell level and Spearman’s rank correlation test were used to calculate their relationship to *SERPINE1* expression (Yuan et al., 2019).

### 2.9 Correlation analysis of *SERPINE1* with immune-associated genes, immune cell infiltration, and cancer-immunity cycle

Based on TCGA in pan-cancer data, the correlation of *SERPINE1* expression with several immunoregulators, infiltration scores of different cells in the tumor microenvironment, and cancer-immunity cycle were evaluated using Pearson’s correlation coefficients. A log_2_ (TPM+1) transformation was performed before analysis. There were four types of immune-associated genes analyzed, including immune checkpoints, chemokines, chemokine receptors, and MHC-related genes. The infiltration scores of different cells in tumor microenvironment were evaluated by seven algorithms, including ESTIMATE (Yoshihara et al., 2013), TIMER (Li et al., 2020), MCP-counter (Becht et al., 2016), CIBERSORT(Newman et al., 2015), quanTIseq (Finotello et al., 2019), xCell (Aran et al., 2017), and EPIC(Racle et al., 2017), using R package “IOBR” (version 0.99.9) (Zeng et al., 2021). The correlation between the expression of *SERPINE1*and marker genes of immune cells, which was obtained from a previous study, was assessed using the “Gene_Corr” module of TIMER2.0 tool (Li et al., 2020; Li et al., 2023). The activity scores of each step of cancer-immunity cycle, which reflects the stepwise events of immune systems’ response to cancer (Chen and Mellman, 2013), were calculated by Tracking Tumor Immunophenotype (TIP, http://biocc.hrbmu.edu.cn/TIP/) (Xu et al., 2018).

### 2.10 Analysis of the relationships of *SERPINE1* expression with TMB, MSI, immunotherapy response, and drug sensitivities

The simple nucleotide variation in pan-cancer data of TCGA annotated by MuTect2 were downloaded from the TCGA GDC (https://portal.gdc.cancer.gov/), and the R package “Maftools” (version 2.8.05) was used to calculate the TMB (Beroukhim et al., 2010; Mayakonda et al., 2018). The MSI in pan-cancer data from TCGA were obtained from a previous study (Bonneville et al., 2017). Cancers with a sample size less than three were eliminated from the TMB and MSI analyses. The relationship between *SERPINE1* expression and immune checkpoint blockade (ICB) therapy response was evaluated by Tumor Immune Syngeneic MOuse (TISMO, http://tismo.cistrome.org/), a syngeneic mouse tumor database for investigation of tumor immunity and immunotherapy response (Zeng et al., 2022). The accuracy of *SERPINE1* in predicting ICB therapy response was further verified by comparing it with other well-known biomarkers in human cohorts based on data from Tumor Immune Dysfunction and Exclusion (TIDE, http://tide.dfci.harvard.edu/) (Fu et al., 2020). Drug sensitivity and mRNA expression data of the NCI-60 cell lines were downloaded from CellMiner (http://discover.nci.nih.gov/cellminer/) (Luna et al., 2021). The relationships of *SERPINE1* expression with TMB, MSI, and drug sensitivities were evaluated using Pearson’s correlation test and the differences between groups are statistically evaluated by Wald test using DESeq2 (Love et al., 2014).

### 2.11 Validating the expression of *SREPINE1* in tissue samples using qRT-PCR

Total RNA was isolated using the TRIzol reagent (Invitrogen). This was followed by its reverse transcription into cDNA using the HiFiScript cDNA Synthesis Kit (CWBio). Quantitative PCR was then conducted using the SYBR Green method in the LightCycler^®^ 480 System (Roche). The relative expression levels of genes were calculated using the 2^−ΔΔCT^ method, with β-actin as the internal reference gene. The forward and reverse primer sequences for *SERPINE1* were 5′-CTC​ATC​AGC​CAC​TGG​AAA​GGC​A-3′ and 5′-GAC​TCG​TGA​AGT​CAG​CCT​GAA​AC-3′, respectively.

### 2.12 Independent cohorts and proteomic level validation of *SERPINE1* abnormal expression in ccRCC

Six ccRCC cohorts were downloaded from the GEO database to verify the abnormal mRNA expression of *SERPINE1* in ccRCC: GSE14994, GSE17895, GSE53000, GSE53757, GSE68417, and GSE71963. Proteomic data and corresponding clinical data from the CPTAC ccRCC discovery Study (PDC000127) were downloaded from CPTAC (https://pdc.cancer.gov/pdc/) to further explore its expression at the protein level (Clark et al., 2019).

## 3 Results

### 3.1 Aberrant expression of *SERPINE1* in pan-cancer

**FIGURE 2:**
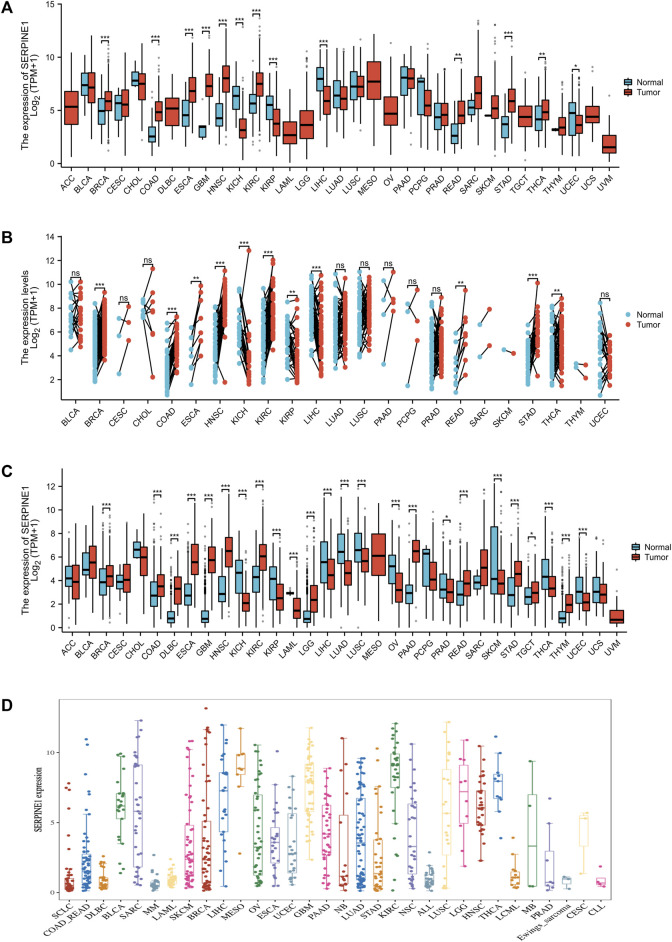
*SERPINE1* expression in pan-cancer. **(A)** The expression of *SERPINE1* in human cancers and normal tissues based on TCGA database. **(B)** The paired analysis of *SERPINE1* expression in human cancers with adjacent normal tissues. **(C)** The differential expression of *SERPINE1* between tumor and normal tissues among cancers based on the integrated data from TCGA and GTEx datasets. **(D)** The expression levels of different cancer cell lines according to CCLE database. *p < 0.05; **p < 0.01; ***p < 0.001.

To explore the expression of *SERPINE1* among cancers, mRNA expression data from the TCGA, GTEx, and CCLE databases were analyzed. Based on the TCGA database alone, the mRNA expression of *SERPINE1* was significantly increased in BRCA, COAD, ESCA, GBM, HNSC, KIRC, READ, STAD, and THCA, but significantly decreased in KICH, KIRP, LIHC, and UCEC (Figure 2A). The paired analysis results were consistent with those of unpaired analysis, except that the result of UCEC was no longer significant (Figure 2B). When analyzed in conjunction with data from the GTEx database, *SERPINE1* expression was also increased in DLBC, PAAD, TGCT, and THYM and decreased in LAML, LUAD, LUSC, OV, PRAD, SKCM, and THYM (Figure 1C). Analysis of data from the CCLE database also indicated that the expression of *SERPINE1* was increased in various cancer types, including BLCA, MESO, GBM, and KIRC, similar to analysis of TCGA data (Figures 2A, D).

### 3.2 *SERPINE1* is enriched in endothelial cells and fibroblasts

**FIGURE 3:**
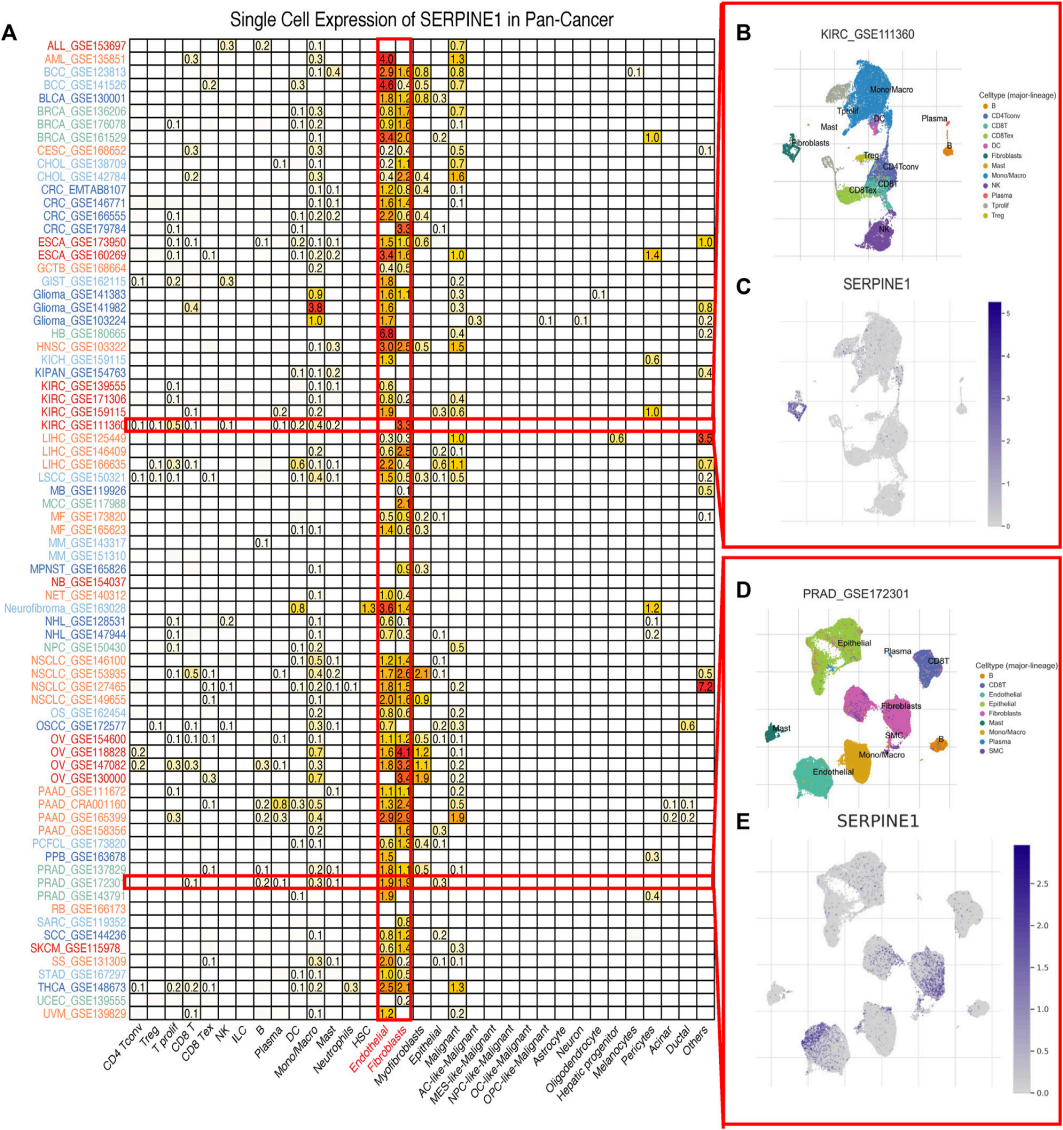
Single cell expression of *SERPINE1* in pan-cancer. **(A)** The expression of *SERPINE1* in different cell types across cancers. **(B)** The distribution of cell types in KIRC GSE111360 cohort. **(C)** The single cell expression of *SERPINE1* in GSE111360 cohort. **(D)** The distribution of cell types in PRAD GSE172301 cohort. **(E)** The single cell expression of *SERPINE1* in GSE 172301 cohort.

Considering the boundedness of bulk RNA-seq, we further investigated the expression of *SERPINE1* at a single-cell level using TISCH2. The results indicated that the expression of *SERPINE1* was enriched in endothelial cells and fibroblasts in most cancer types (Figure 3A). The results for GSE11360 and GSE172301 are shown as examples (Figures 3B–E).

### 3.3 Genetic alteration and DNA methylation analysis of *SERPINE1* in pan-cancer

**FIGURE 4:**
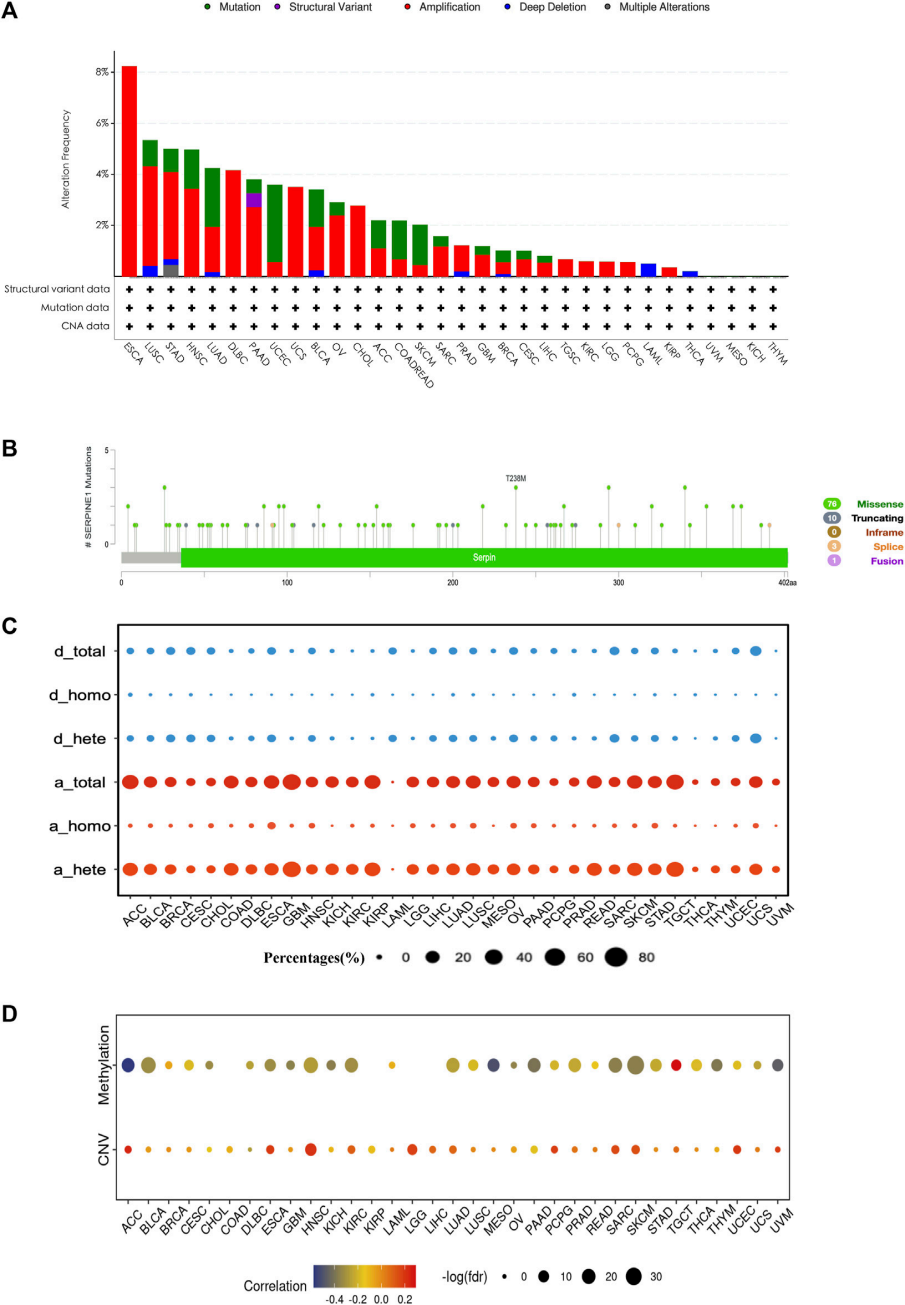
The genetic alteration and DNA methylation profile of *SERPINE1* in pan-cancer. **(A)** The genetic alteration condition of *SERPINE1* among cancers based on cBioPortal database. **(B)** The composition of *SERPINE1* mutation in pan-cancer according to cBioPortal database. **(C)** The composition of *SERPINE1* copy number variation according to GSCA database. d_total, total copy number deletion percentage; d_homo, homozygous copy number deletion percentage; d_hete, heterozygous copy number deletion percentage; a_total, total copy number amplification percentages percentage; a_homo, homozygous copy number amplification percentage; a_hete, heterozygous copy number amplification percentage. **(D)** Spearman’s rank correlations of *SERPINE1* expression with the DNA methylation level and copy number variance.

Considering that the mRNA expression level of a gene can be influenced by epigenetics, we investigated the genetic alteration and DNA methylation level of *SERPINE1* using cBioPortal and GSCA datasets. Amplification is the most common type of genetic alteration in most cancers, dominated by heterozygous amplification, followed by mutation, dominated by missense mutation (Figures 4A–C). Correlation analysis showed that the expression of *SERPINE1* was negatively correlated with the DNA methylation level and positively correlated with the DNA copy number in most cancer types (Figure 4D).

### 3.4 *SERPINE1* is a prognostic and diagnostic factor for various cancers

**FIGURE 5:**
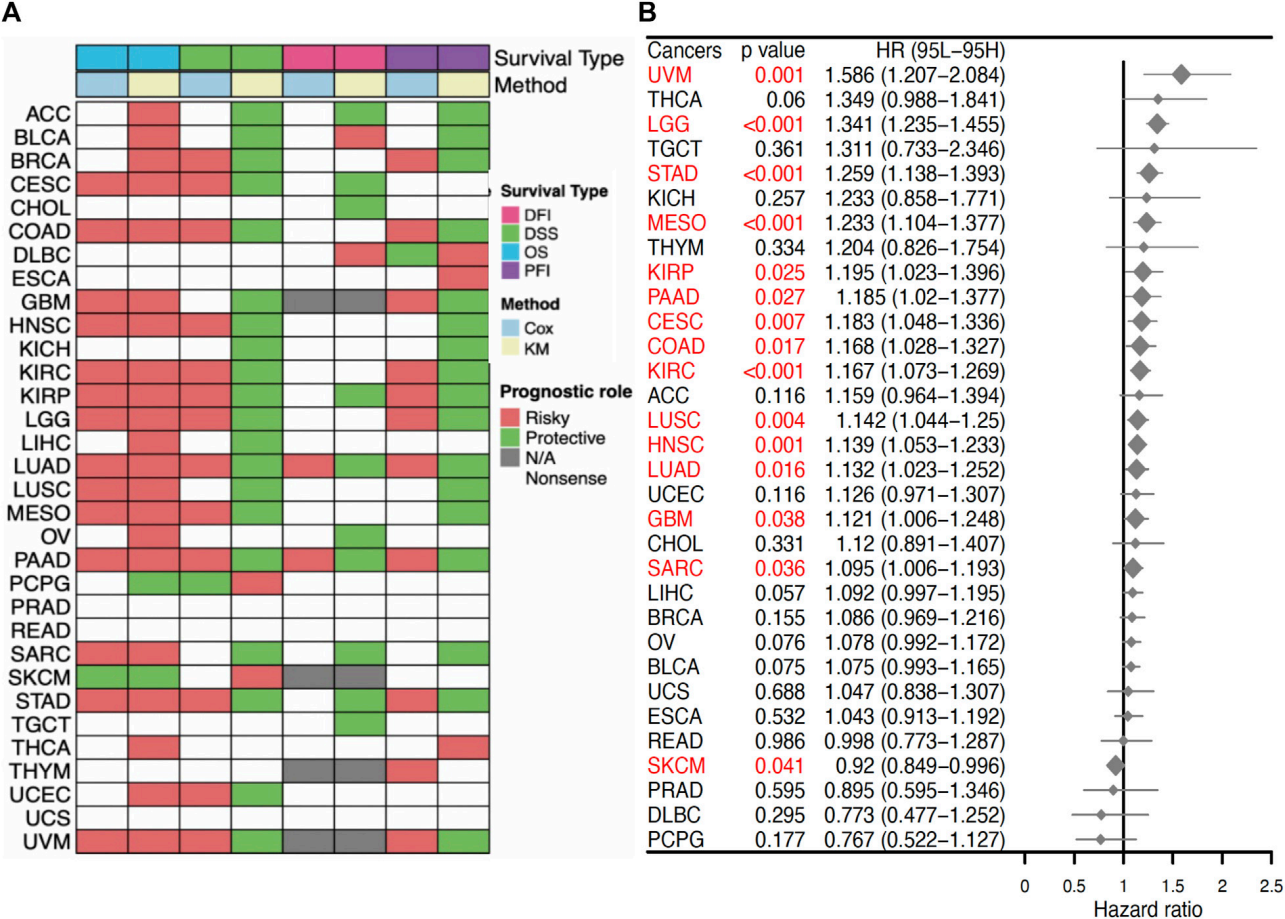
Prognostic value of *SERPINE1* in pan-cancer. **(A)** Correlations of *SERPINE1* expression with overall survival (OS), disease-specific survival (DSS), disease-free interval (DFI) and progression-free interval (PFI) based on univariate Cox regression and Kaplan-Meier method. Red indicates a risky role of *SERPINE1* for prognosis, while green represents a protective role. **(B)** The prognostic role of *SERPINE1* expression to patients’ overall survival (OS) in cancers by univariate Cox regression. Significant results (*p* < 0.05) are highlighted in red.

To evaluate the prognostic capacity of *SERPINE1* in cancers, both univariate Cox regression and Kaplan–Meier analysis were performed. *SERPINE1* was found to significantly reduce overall survival in ACC, BLCA, BRCA, CESC, COAD, GBM, HNSC, KIRC, KIRP, LGG, LIHC, LUAD, LUSC, MESO, OV, PAAD, SARC, STAD, THCA, UCEC, and UVM, while it was found to play a protective role for overall survival in PCPG and SKCM (Figure 5A). Besides, it is noteworthy that *SERPINE1* was associated with poor prognosis for all four types of prognostic outcomes in LUAD and PAAD (Figure 5A). Univariate Cox regression results for the OS of all cancers are shown in Figure 5B. These findings suggested the general prognostic value of *SERPINE1* expression in various cancers. The diagnostic value of *SERPINE1* was examined using ROC curve as well. As shown in Supplementary Figure S1, SERPINE1 showed high diagnostic value in 11 kinds of cancers (AUC > 0.7), including CHOL, COAD, ESCA, GBM, HNSC, KICH, KIRC, KIRP, LIHC, READ, and STAD, indicating its crucial role in cancer diagnosis.

### 3.5 *SERPINE1* is associated with the immune response and tumor malignancy in pan-cancer

**FIGURE 6:**
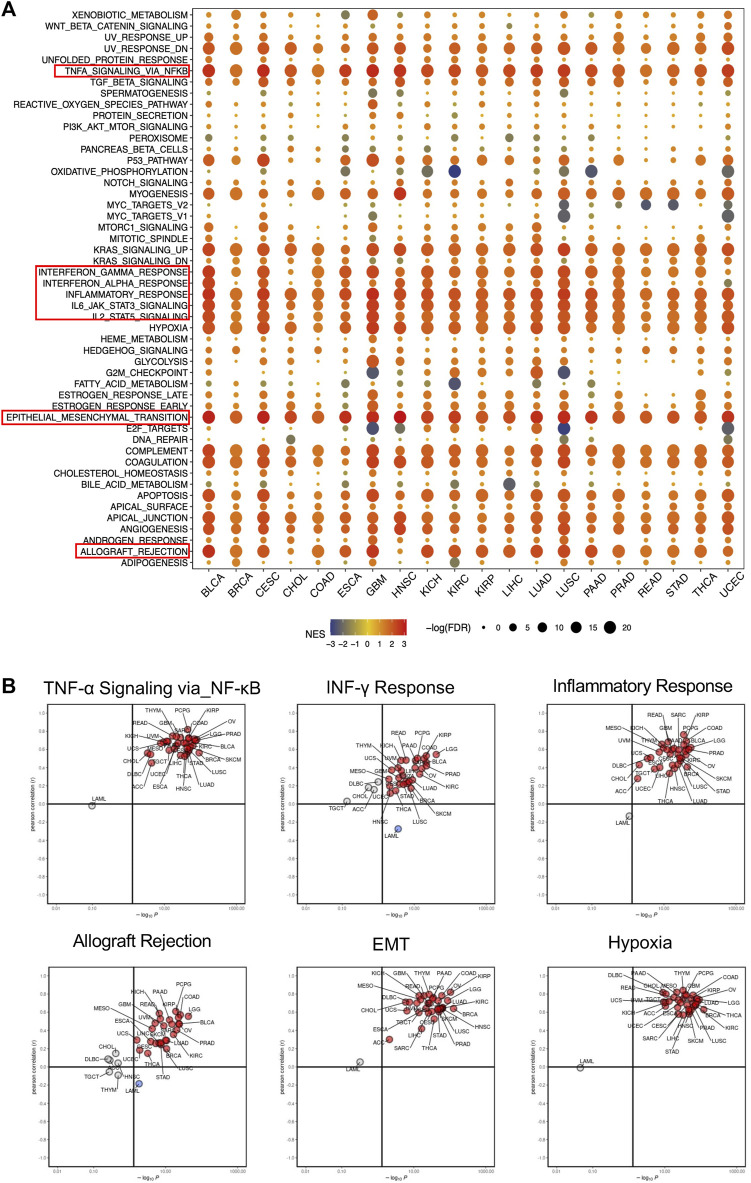
Gene function analysis of *SERPINE1* in pan-cancer based on GSEA and ssGSEA. **(A)** The hallmarks gene set enrichment analysis (GSEA) of *SERPINE1* in pan-cancer. The size of circle represents the FDR value of enrich term in each cancer, and the color indicates the normalized enrichment score (NES) of each term. **(B)** The single sample gene set enrichment analysis (ssGSEA) of *SERPINE1* in pan-cancer. Cancer types with significant positive correlations (*p* < 0.05) with ssGSEA scores are highlighted in red, while those with significant negative correlations (*p* < 0.05) are highlighted in blue.

**FIGURE 7:**
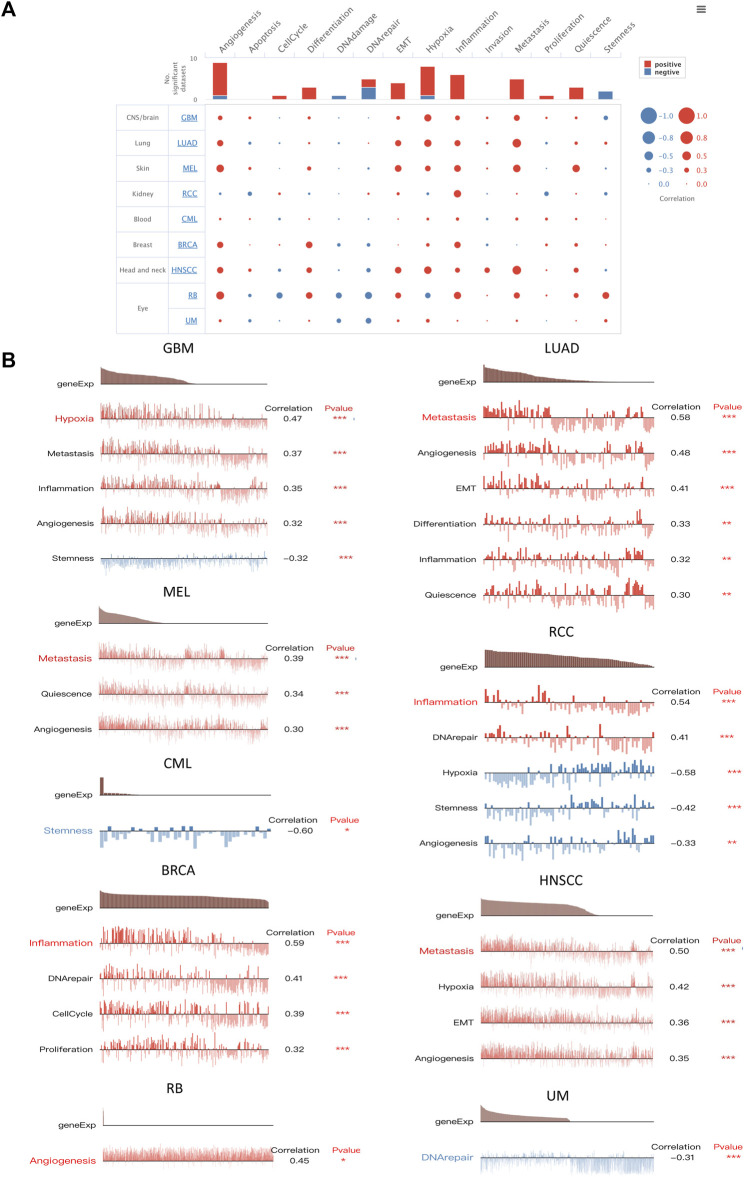
Functional relevance analysis of *SERPINE1* in single-cell resolution by CancerSEA. **(A)** The association between *SERPINE1* expression and single-cell states in cancers. **(B)** Single-cell states that significantly associated with each cancer. *p < 0.05; **p < 0.01; ***p < 0.001.

To better understand the biological roles of *SERPINE1*, we evaluated the enrichment of pathways associated with *SERPINE1* expression using GSEA at the bulk-RNA level and CancerSEA at the single-cell level. The GSEA results revealed that a number of immune-related pathways were enriched in samples with high *SERPINE1* expression in most cancers, including TNF-α signaling via NF-κB, IFN-γ response, IFN-β response, inflammation response, IL-6-JAK-STAT3 signaling, IL-2-STATA5 signaling, and complement and allograft rejection, indicating a potential relationship between *SERPINE1* and cancer immunity (Figure 6A). The results of ssGSEA for selected gene sets were consistent with those of GSEA (Figure 6B). The results from CancerSEA demonstrated that *SERPINE1* was positively related with angiogenesis, hypoxia, inflammation, and metastasis in most cancers and negatively related with DNA damage, DNA repair, and stemness in some cancers (Figures 7A, B), which were consistent with the abovementioned results. Based on these results, we speculated that *SERPINE1* may regulate tumor progression by influencing immune-related processes within tumors.

### 3.6 *SERPINE1* expression is correlated with the expression of various immunoregulators in pan-cancer

**FIGURE 8:**
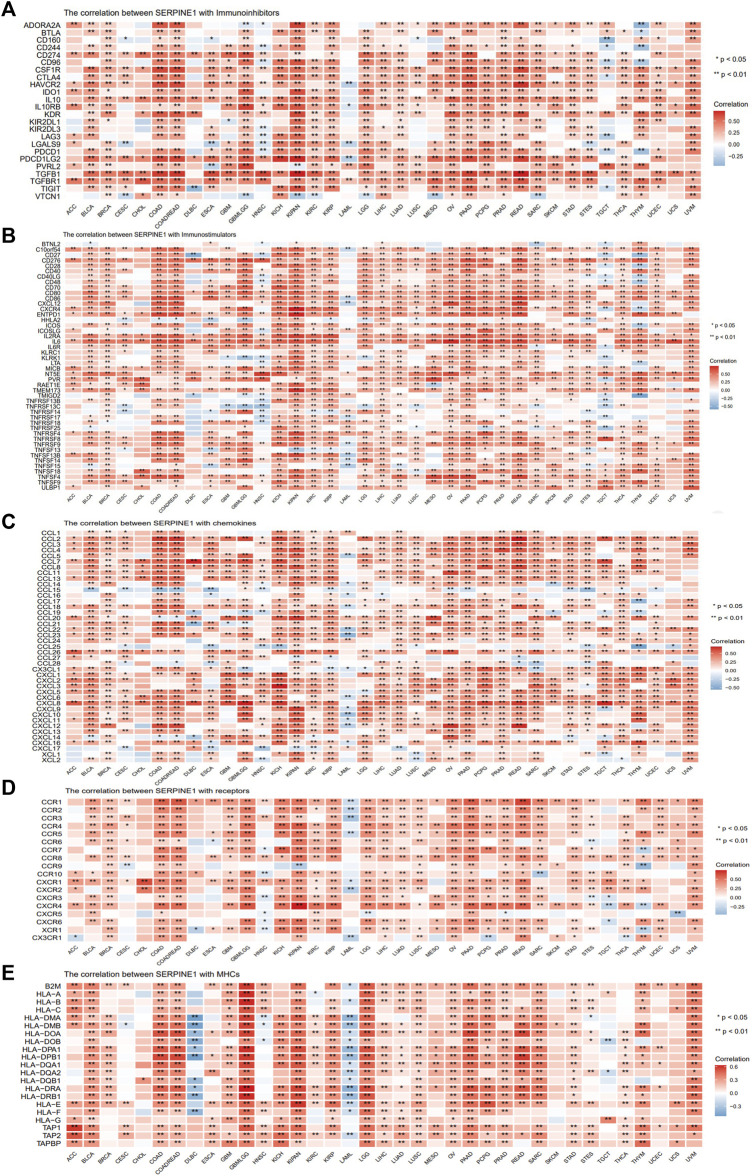
Co-expression analysis of *SERPINE1* with immunoregulators in pan-cancer. **(A)** The Pearson’s correlation of *SERPINE1* with inhibitory immune-checkpoints genes in cancers. **(B)** The Pearson’s correlation of *SERPINE1* with stimulatory immune-checkpoints genes in cancers. **(C)** The Pearson’s correlation of *SERPINE1* with chemokines. **(D)** The Pearson’s correlation of *SERPINE1* with chemokine receptors **(E)** The Pearson’s correlation of *SERPINE1* with MHC-related genes in cancers. *p < 0.05; **p < 0.01; ***p < 0.001.

Given the well-known roles of immunoregulators in cancer immunity, the relationships between *SERPINE1* and immune checkpoints, chemokines, chemokine receptors, and MHC-related genes were analyzed. The results demonstrated that *SERPINE1* expression was positively correlated with the expression of most inhibitory immune checkpoints, including *CD274* (PD-L1), *PDCD1* (PD-1), *CTLA4,* and *HAVCR2* (TIM-3) in most cancers, especially GBM-LGG, KIPAN, UVM, PAAD, and COAD-READ (Figure 8A). Moreover, expression of *SERPINE1* was found to be significantly associated with expressions of various immunostimulators, chemokines, chemokine receptors and MHC-related genes in pan cancer as well (Figures 8B–E).

### 3.7 *SERPINE1* expression is correlated with immune cell infiltration and cancer-immunity cycle in pan-cancer

**FIGURE 9:**
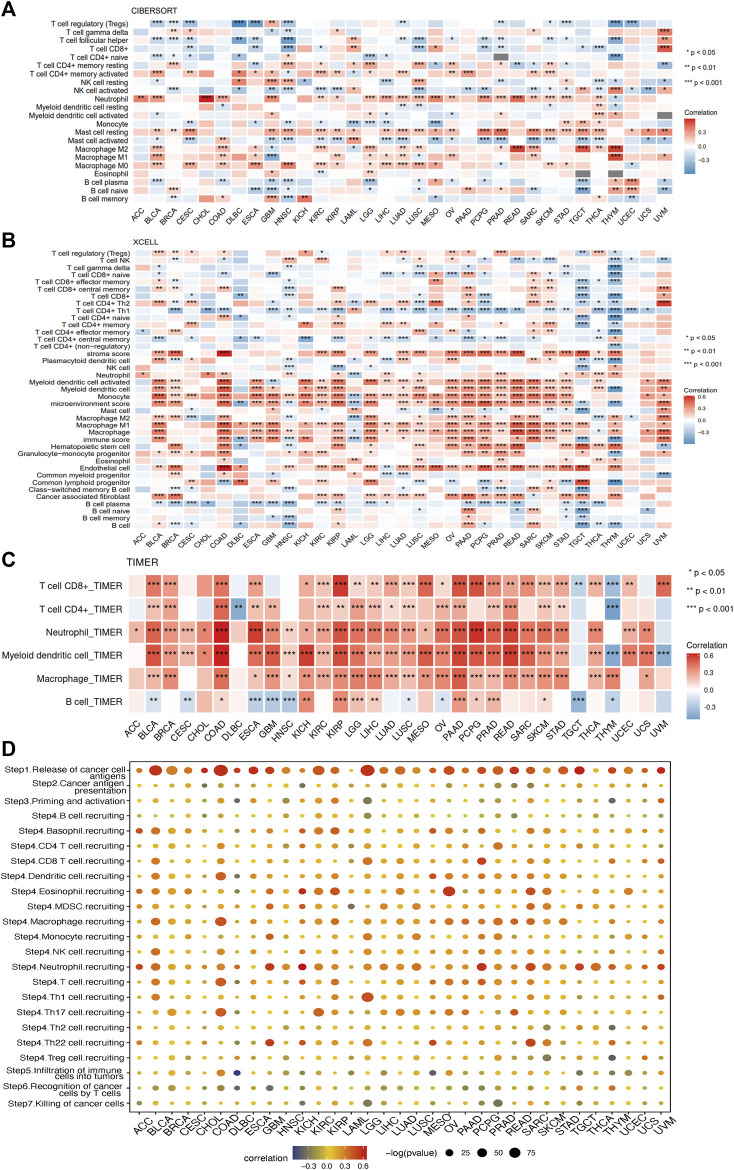
Correlations of SERPINE1 expression with immune cells infiltrations in pan-cancer. **(A)** The correlations of *SERPINE1* expression with infiltration of 22 types of immune cells in cancers based on CIBERSORT algorithm. **(B)** The correlations of *SERPINE1* expression with infiltration of 64 types of immune and stromal cell in cancers based on xCell algorithm. **(C)** The correlations of *SERPINE1* expression with infiltration of six types of immune cell in cancers based on TIMER algorithm. **(D)** Correlation between SERPINE1 expression and cancer-immunity cycle. The size of circle represents the *p*-value of correlation test, and the color indicates the Pearson’s correlation coefficient. *p < 0.05; **p < 0.01; ***p < 0.001.

To gain further insight into the relationship between *SERPINE1* and cancer immunity, we conducted immune cell infiltration analysis using different algorithms across a range of cancers. All algorithms showed that *SERPINE1* expression was significantly correlated with the infiltration scores of multiple immune cells, although there was some variation in the results for specific cell types. The results from ESTIMATE indicated that *SERPINE1* expression was positively correlated with both the immune score and stromal score in most cancers, especially KIPAN, GBM-LGG, and COAD-READ. Notably, no significantly negative relationship was found between *SERPINE1* expression and stromal score in all cancers (Supplementary Figure S2). According to the CIBERSORT algorithm, *SERPINE1* expression was negatively correlated with the infiltration of plasma cells, memory B cells, naïve CD4^+^ T cells, CD8^+^ T cells, follicular helper T cells, regulatory T cells, activated NK cells, and resting mast cells and positively correlated with the infiltration of activated memory CD4^+^ T cells, macrophages, activated mast cells, and neutrophils in most cancers (Figure 9A). As for the xCell algorithm, its results indicated that *SERPINE1* expression was negatively correlated with the infiltration of CD4^+^ Tcm cells, NKT cells, plasma cells, and Th1 cells and positively correlated with the infiltration of fibroblasts, macrophages, neutrophils and regulatory T cells (Figure 9B). The results from TIMER algorithm revealed that *SERPINE1* expression was positively correlated with the infiltration of all cell types, except B cells which did not reach significance in most cancers (Figure 9C). The results of MCP-counter algorithm were similar with that of TIMER algorithm, except that the infiltration of T cells showed significant negative correlation with *SERPINE1* expression in 10 types of cancers (Supplementary Figure S3A). The results of the quanTIseq algorithm indicated that *SERPINE1* expression was positively correlated with M1 macrophages, M2 macrophages, neutrophils and regulatory T cells (Supplementary Figure S3B). The results of the EPIC algorithm showed that *SERPINE1* expression was positively correlated with the infiltration of cancer associated fibroblasts (CAFs), endothelial cells, and macrophages, and negatively correlated with the infiltration of CD8^+^ T cells (Supplementary Figure S3C). We also analyzed the correlation between the expression of *SERPINE1* and immune cells’ marker genes. The results showed the expression of SERPINE1 was positively correlated with that of most immune cells’ marker genes in most cancer, especially the maker genes of neutrophils, monocyte, tumor-associated macrophages (TAMs), M2 macrophages, and regulatory T cells (Supplementary Figure S4). These results suggested that *SERPINE1* may regulate the immune response of cancers.

The relationship between *SERPINE1* expression and the cancer-immunity cycle, which can reflect the stepwise events in anticancer immune response, was analyzed as well. As shown in Figure 8D, high expression of *SERPINE1* was positively correlated with the activities of step 1 and step 4, which represents the release of cancer cell antigen and the recruiting of immune cells respectively, but negatively correlated with the activities of step 5, 6 and 7, which represents the infiltration of immune cells into tumors, the recognition of cancer cells by T cells, and the killing of cancer cells, in lots of cancer types. These results provide further evidence that *SERPINE1* may be involved in the regulation of the immune response to cancer.

### 3.8 *SERPINE1* is associated with TMB, MSI, immunotherapy response, and drug sensitivity in pan-cancer

**FIGURE 10:**
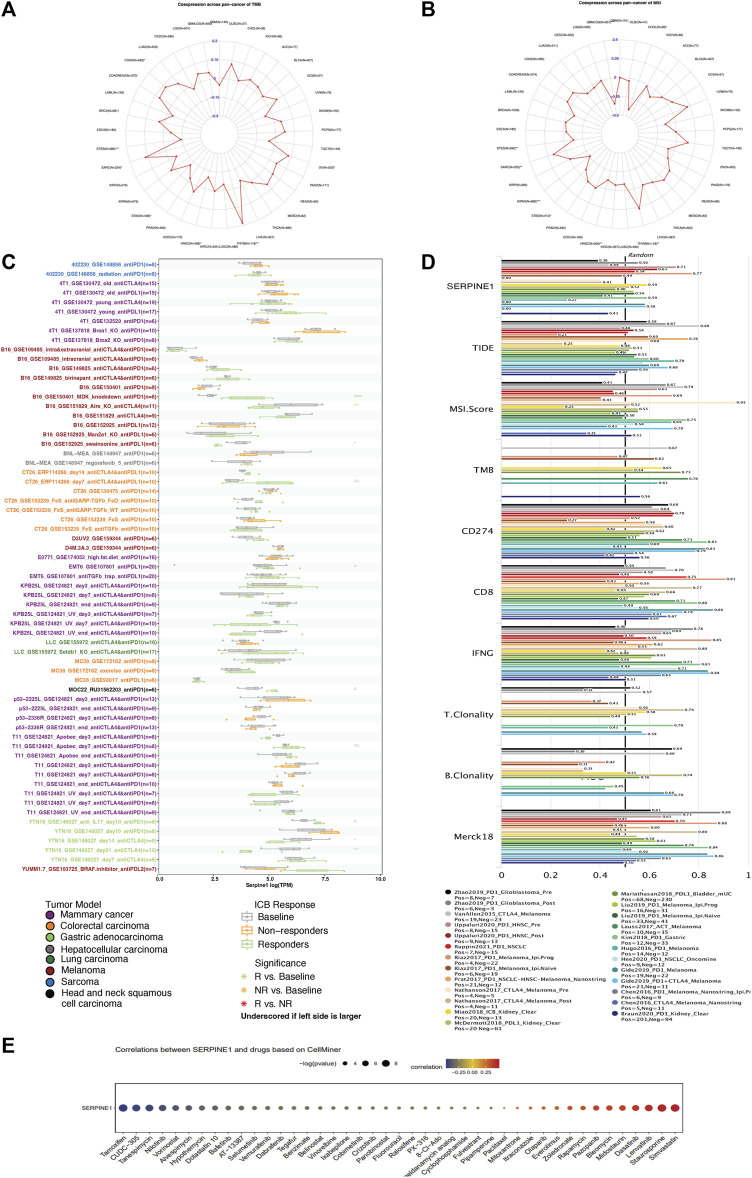
Relevance analysis of *SERPINE1* expression with tumor mutation burden (TMB), microsatellite instability (MSI), immunotherapy response, and drug sensitivity. **(A)** The correlation of *SERPINE1* with TMB in pan-cancer. **(B)** The correlation of *SERPINE1* with MSI in pan-cancer. **(C)** Expression of *SERPINE1* in mice with different responses to immune checkpoint blockade (ICB) therapy based on data from TISMO database. **(D)** The area under the receiver operating characteristic curve (AUC) of *SERPINE1* and other biomarkers in predicting ICB therapy response. **(E)** The association of *SERPINE1* expression with drug sensitivity based on CellMiner database. *p < 0.05; **p < 0.01; ***p < 0.001.

Due to the predictive value of TMB and MSI for immunotherapy response, the relationships between TMB, MSI, and *SERPINE1* expression were evaluated. *SERPINE1* expression was found to be positively associated with TMB in THYM, COAD, COADREAD, SARC, and OV and negatively correlated with STES, STAD, and HNSC (Figure 10A). In addition, *SERPINE1* expression levels in seven cancers were also significantly correlated with MSI in GBM-LGG, KIPAN, STES, HNSC, STAD, CHOL, SARC and THYM (Figure 10B). Then, we directly analyzed the relationship between *SERPINE1* expression and immunotherapy response. As shown in Figure 10C, *SERPINE1* expression could significantly predict the immunotherapy response in nine murine cohorts, with responders showing elevated *SERPINE1* expression levels in six cohorts and decreased *SERPINE1* expression levels in three cohorts. We also compared the predictive power of *SERPINE1* to ICB response with other biomarkers in human cohorts. The area under curve (AUC) of *SERPINE1* was above 0.5 in 12 cohorts and above 0.7 in two cohorts, which is similar to T clonality but lower than other classical biomarkers such as MSI, TMB, and CD274 (Figure 10D). These results indicated that *SERPINE1* could predict the response of cancer patients to immunotherapy to some extent, though its predictive power might be lower than that of some classical markers. Furthermore, the correlations between *SERPINE1* expression and drug sensitivities were explored using CellMiner. We found that *SERPINE1* expression was positively correlated with sensitivities towards 13 drugs, including simvastatin, staurosporine, and pazopanib, and negatively correlated with sensitivities towards 30 drugs, including tamoxifen, tanespimycin, and nilotinib (Figure 10E).

### 3.9 Elevated expression of *SERPINE1* is associated with unfavorable prognosis in patients with ccRCC

**FIGURE 11:**
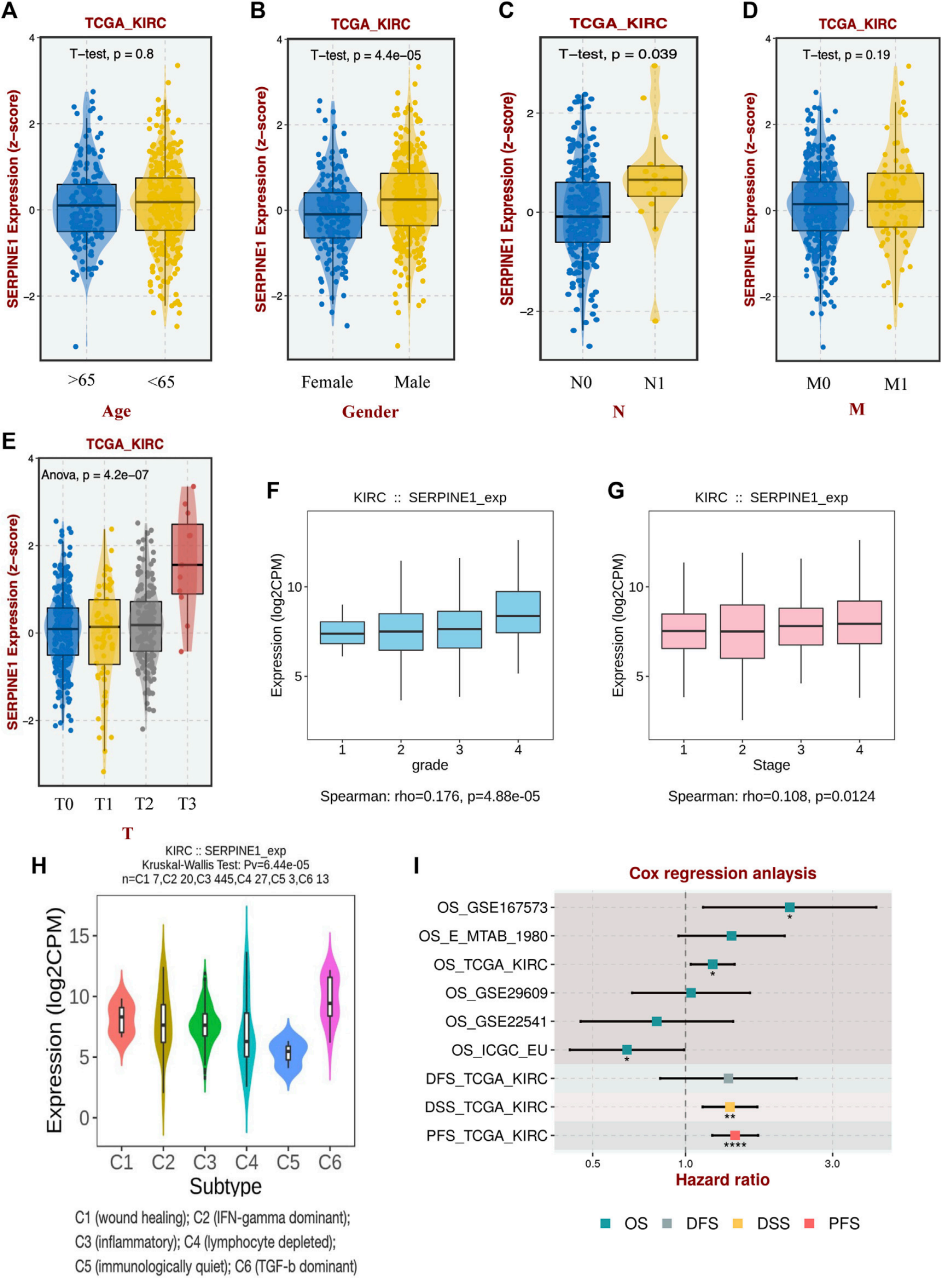
Association between *SERPINE1* and clinicopathological characteristics of clear cell renal cell carcinoma. **(A)** The expression of *SERPINE1* in patients of different ages. **(B)** The expression of *SERPINE1* in patients of different genders. **(C)** The expression of *SERPINE1* in patients of different lymph node metastasis statuses. **(D)** The expression of *SERPINE1* in patients of different remote metastasis statuses. **(E)** The expression of *SERPINE1* in patients of different primary tumor stages. **(F)** The expression of *SERPINE1* in different histological grades. **(G)** The expression of *SERPINE1* in different cancer stages. **(H)** The expression of *SERPINE1* in different immune subtypes. **(I)** The correlation of *SERPINE1* with prognosis based on different datasets. OS, overall survival; DFS, disease-free survival; DSS, disease-specific survival; PFS, progression-free survival; *p < 0.05; **p < 0.01; ***p < 0.001.

Given the limited availability of reports on the role of *SERPINE1* in ccRCC, we investigated the role of *SERPINE1* in ccRCC. Our findings revealed that *SERPINE1* expression is associated with male sex, lymph node metastasis, higher T stage, higher histological grade, higher pathological stage, and different immune subtypes (Figures 11A–H). Cox regression analyses revealed upregulated *SERPINE1* expression as a risk factor for OS, DSS, and PFS using data from the TCGA database and as a risk factor for OS using GSE167573 cohort data from the GEO database (Figure 11I). However, the result of Cox regression using RECA-EU project data from ICGC database indicated upregulated *SERPINE1* expression as a protective factor for OS (Figure 11I). The multivariate Cox regression was performed based on TCGA database, and the results verified the independence of *SERPINE1*’s prognostic value for patient with ccRCC (Supplementary Figure S5).

### 3.10 *SERPINE1* expression is associated with the tumor microenvironment in clear cell renal cell carcinoma

**FIGURE 12:**
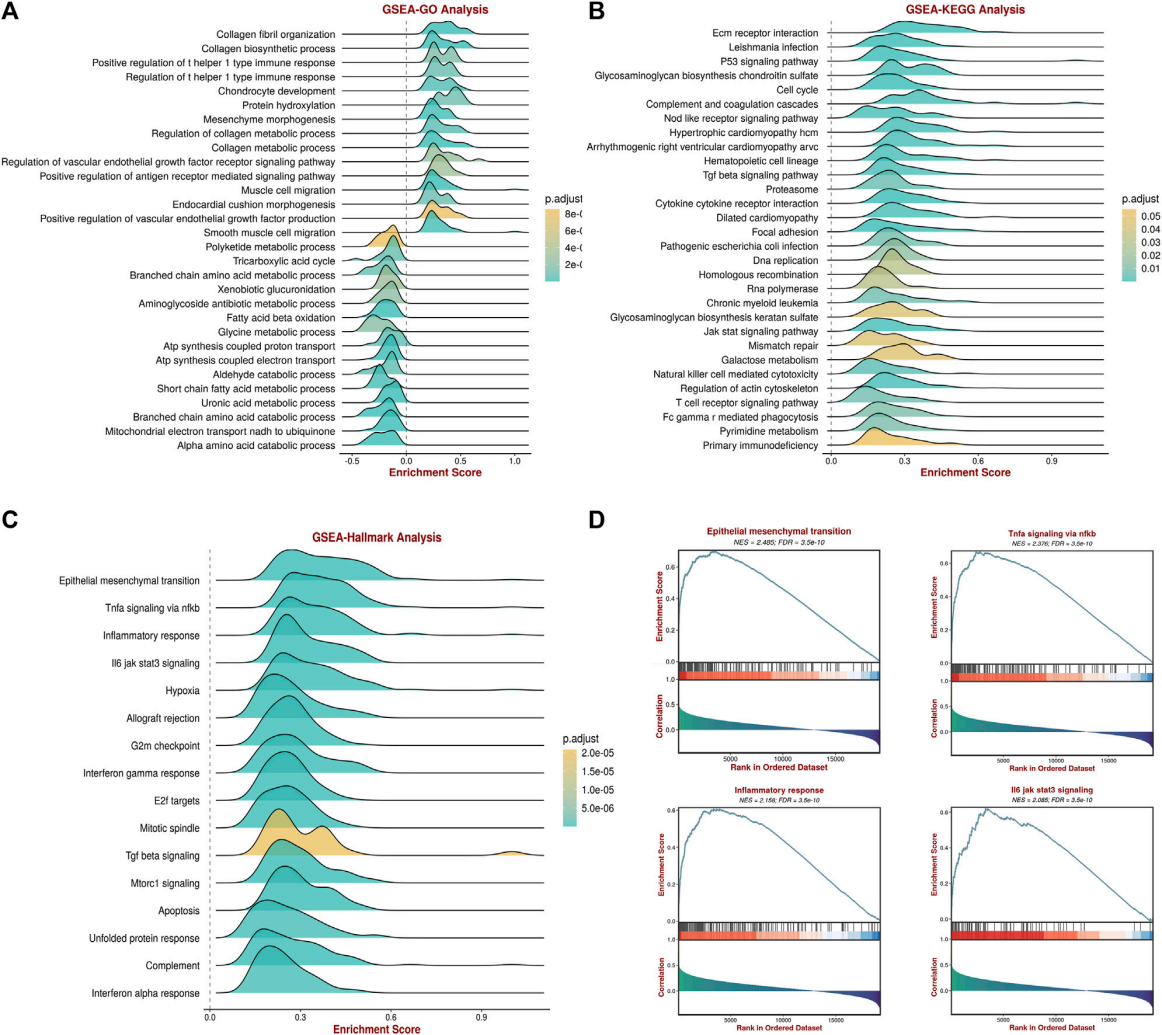
Gene set enrichment analysis (GSEA) of *SERPINE1* in clear cell renal cell carcinoma (ccRCC). **(A)** The associations of *SERPINE1* expression with several Gene Ontology (GO) terms. **(B)** The associations of *SERPINE1* expression with several Kyoto Encyclopedia of Genes and Genomes (KEGG) pathways. **(C)** The associations of *SERPINE1* expression with several Hallmark terms. **(D)** The GSEA results of certain Hallmark terms.

To further investigate the role of *SERPINE1* in ccRCC, gene set enrichment analysis was performed. The results revealed that *SERPINE1* expression was significantly related with various biological processes. Further, GO analysis showed that *SERPINE1* expression was positively correlated with collagen fibril organization, collagen biosynthetic process, and the regulation of T helper 1 type immune response, while it was negatively correlated with metabolism-related processes, such as tricarboxylic acid cycle, fatty acid beta oxidation, and the alpha amino acid catabolic process (Figure 12A). In the KEGG pathway analysis, several malignancy-related pathways, such as the P53 signaling pathway, cell cycle and DNA replication pathways, as well as immunity-related pathways, such as natural killer cell mediated cytotoxicity and T cell receptor signaling pathways, were found to be significantly positively correlated with *SERPINE1* expression (Figure 12B). Similar results were obtained through the hallmark analysis (Figures 12C, D). These findings revealed that *SERPINE1* may be involved in the formation of the tumor microenvironment in ccRCC and were consistent with the results in pan-cancer.

### 3.11 Validation of *SERPINE1* expression in tumor tissues using qRT-PCR, independent cohorts, and proteomic data

**FIGURE 13:**
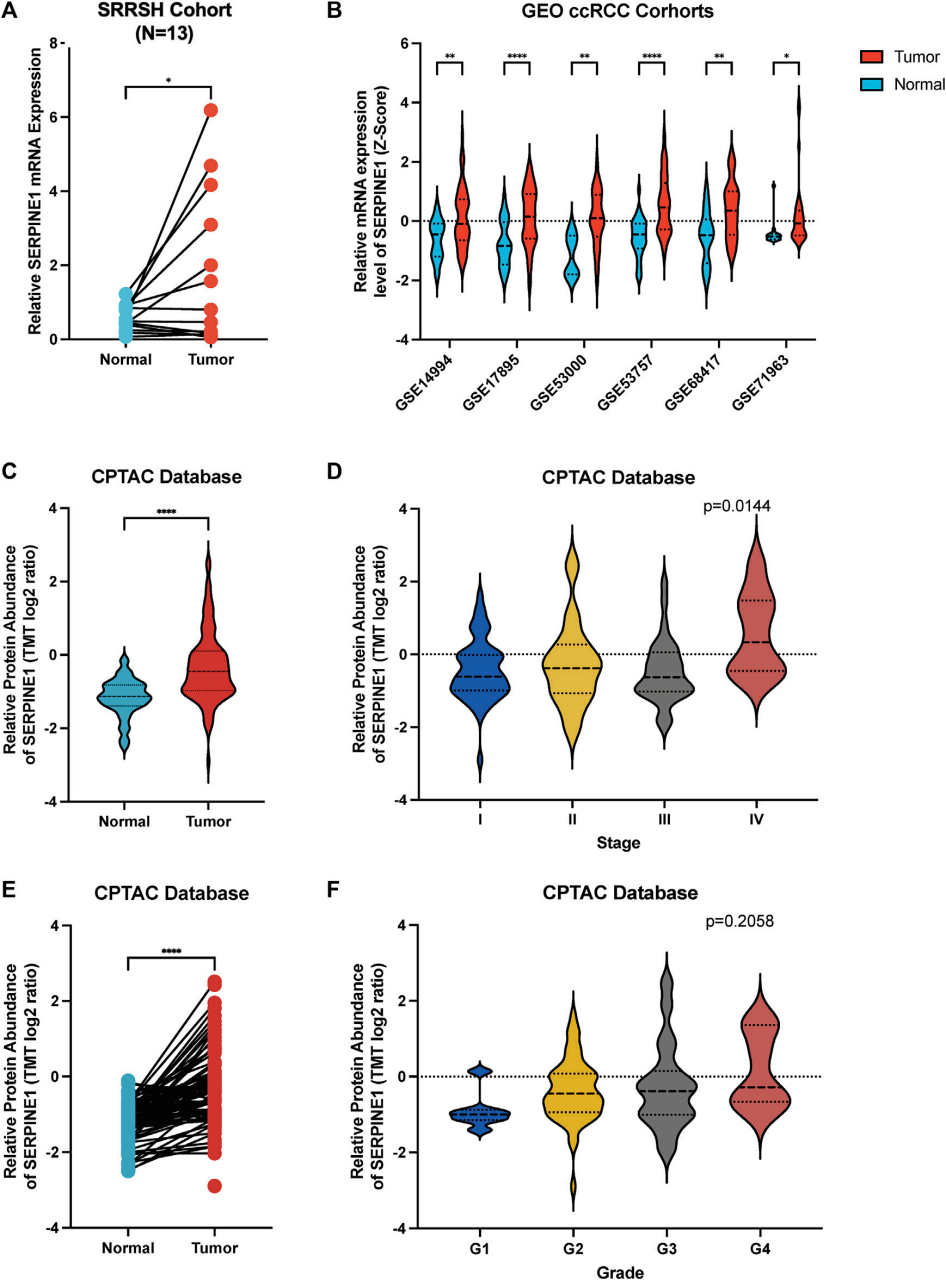
Validation of aberrant expression of *SERPINE1* in ccRCC by qRT-PCR, independent cohorts and proteome. **(A)** qRT-PCR analysis of *SERPINE1* expression in clear cell renal cell carcinoma and paired adjacent normal tissues based on patient samples from Sir Run Run Shaw Hospital. **(B)** Analysis of 6 GEO cohorts regarding *SERPINE1* expression in ccRCC and normal samples. **(C)** Unpaired analysis of *SERPINE1* protein abundance in ccRCC and adjacent normal tissue based on CPTAC database. **(D)** The SERPINE1 protein abundance in patients with different stages of ccRCC. **(E)** Paired analysis of SERPINE1 protein abundance in ccRCC and adjacent normal tissue based on CPTAC database. **(F)** The SERPINE1 protein abundance in patients with different histological grades of ccRCC *p < 0.05; **p < 0.01; ***p < 0.001.

Finally, we confirmed the aberrant expression of *SERPINE1* in ccRCC samples. Our ccRCC patient samples and six independent GEO cohorts exhibited significantly increased levels of *SERPINE1* expression, as expected (Figures 13A, B). Additionally, analysis of CPTAC data revealed that *SERPINE1* expression was also elevated at the protein level in ccRCC samples (Figures 13C–F), which further enhanced the credibility of our study findings.

## 4 Discussion

*SERPINE1*, a regulator of the fibrinolytic system, was found to be associated with tumor progression and metastasis in several cancers; however, its detailed mechanisms of action in various cancers remain obscure (Hanekom et al., 2002; Becker et al., 2010; Duffy et al., 2014; Mashiko et al., 2015; Jevrić et al., 2019; Sotiropoulos et al., 2019). In the present research, we performed a multi-omics integrated analysis to explore the expression, prognostic value, and possible underlying mechanisms of action of *SERPINE1* in pan-cancer.

Expression analysis based on the TCGA and GTEx databases indicated the aberrant expression of *SERPINE1* in several cancers. Overexpression of *SERPINE1* and its correlation with poor prognosis has been reported in several cancers (Hanekom et al., 2002; Becker et al., 2010; Duffy et al., 2014; Nakatsuka et al., 2017; Jevrić et al., 2019; Sotiropoulos et al., 2019). The results of the present study confirmed the prevalence of aberrant *SERPINE1* expression among cancers. Given the bulk RNA sequencing data is the average expression of different cells, which will lead to the loss of information about cells heterogeneity, we analyzed the expression of *SERPINE1* at single-cell level as well, and the results showed that *SERPINE1* expression was enriched in the endothelial cells and fibroblasts, consistent with the findings of a previous study, indicating the functions of *SERPINE1* might be related with these two cell types, such as angiogenesis and regulating TME (Placencio and DeClerck, 2015; Clark et al., 2019; Chen et al., 2021b; de Visser and Joyce, 2023). Subsequently, we analyzed possible reasons for the aberrant expression of *SERPINE1* in cancers. DNA methylation level and DNA copy number variation are well-known ways to influence the gene expression, thus their correlation with *SERPINE1* was analyzed (Morgan et al., 2018; Pös et al., 2021). *SERPINE1* expression showed a positive correlation with copy number variance and a negative correlation with the DNA promoter methylation level in most cancers, suggesting its potential role in CNV and the DNA methylation level. Some studies have indicated that some microRNAs and long noncoding RNAs are involved in the regulation of *SERPINE1* expression (Tan et al., 2021; Teng et al., 2021; Zhao and Liu, 2021). Moreover, transforming growth factor β (TGF-β) has been implicated in the regulation of *SERPINE1* expression (Kutz et al., 2001; Ma et al., 2002), and a positive correlation of TGF-β expression with *SERPINE1* expression was observed through the GSEA analysis conducted in the present study.

Next, we explored the relationship between *SERPINE1* expression and the prognosis of cancer patients. High *SERPINE1* expression was found to be a risk factor for overall survival in several cancers, consistent with the findings of previous studies (Hanekom et al., 2002; Becker et al., 2010; Duffy et al., 2014; Nakatsuka et al., 2017; Jevrić et al., 2019; Sotiropoulos et al., 2019). Hence, we speculated that *SERPINE1* may be a prognostic biomarker for various cancers.

To further explore the mechanisms underlying the role of *SERPINE1* in cancers, we performed GSEA on pan-cancer data from TCGA. Several immune-related pathways were found to be significantly associated with high *SERPINE1* expression in most cancers, including TNF-α signaling via NF-κβ, INF-γ response, and inflammation response. Similar results were also found in the single cell-level analysis performed by us using CancerSEA. Therefore, we believe that *SERPINE1* is involved in cancer immunity and tumor malignancy (Iwaki et al., 2012; Declerck and Gils, 2013; Placencio and DeClerck, 2015; Chen et al., 2021; Sillen and Declerck, 2021).

Since immunoregulators are known to be important for the immune response, we next performed a co-expression analysis of *SERPINE1* to further understand its roles in cancer immunity. The results demonstrated that *SERPINE1* expression was positively and significantly correlated with the expression of immune-checkpoints, chemokines, chemokine receptors, and MHC-related genes in most cancers, indicating the remarkable effect of *SERPINE1* on the immune system and consistent with previous studies about the pro-inflammatory and pro-angiogenesis roles of *SERPINE1* (Iwaki et al., 2012; Declerck and Gils, 2013; Placencio and DeClerck, 2015). Traditionally, CD8^+^ T cells, memory B cells, plasma cells, follicular helper T cells, activated NK cells, NKT cells, and M1 macrophages are thought to be anti-cancer cells in the tumor microenvironment (TME), while regulatory T cells, M2 macrophages, and cancer associated fibroblasts are considered to be pro-cancer cells (Fridman et al., 2012; Sica and Mantovani, 2012; Bae et al., 2019; Chen and Song, 2019; Togashi et al., 2019; St Paul and Ohashi, 2020; Laskowski et al., 2022; Laumont et al., 2022; Li H. et al., 2023; Cai et al., 2023; Gutiérrez-Melo and Baumjohann, 2023). Therefore, we speculated that high *SERPINE1* expression may play an immunosuppressive role in the tumor microenvironment due to its inverse correlation with several anti-cancer cells and positive correlation with several pro-cancer cells. The result of cancer-immunity cycle analysis further validated our speculation. Although *SERPINE1* expression was positive correlated with the recruiting of most immune cells, the infiltration of immune cells into tumors, recognition of cancer cells by T cells, and killing of cancer cells were found to be negatively correlated with *SERPINE1* expression in lots of cancers. However, given that our bioinformatic analysis based on bulk-RNA sequencing data has several limitations, further investigations are warranted.

Next, we validated the abnormal expression of *SERPINE1* and its potential biological functions in clear cell renal cell carcinoma. Upregulated *SERPINE1* expression was found to be associated with several clinical features of ccRCC, such as lymph node metastasis, high T stage, high histological grade, and high pathological stage. As for the gene function analysis performed using GSEA, collagen-associated processes, immune-associated processes, and malignancy-related pathways were found to be positively correlated with *SERPINE1* expression in ccRCC. These findings are consistent with our speculation that *SERPINE1* expression is involved in the regulation of the tumor microenvironment. Next, we further explored *SERPINE1* expression in ccRCC in clinical patient samples and independent datasets.

Given the role of abnormal *SERPINE1* expression in cancer development, it is important to consider how to interfere its expression to benefit cancer patients. As PAI-1 is a well-known regulator of the plasminogen activation system, many efforts have been devoted to the development of selective PAI-1 inhibitors (Fortenberry, 2013; Placencio et al., 2015; Sillen and Declerck, 2020; 2021). Some marketed drugs, including insulin-sensitizing agents, angiotensin-converting enzyme inhibitors (ACEI), and statins, have shown the ability to attenuate the synthesis or secretion of *SERPINE1* (Brown et al., 2002b; Ersoy et al., 2008; Baluta and Vintila, 2015). Specifically, insulin resistance has been found to be associated with elevated plasma PAI-1 levels (Juhan-Vague and Alessi, 1997), and both proinsulin and insulin can stimulate PAI-1 expression (Sakamoto et al., 1999; Nordt et al., 2001), thus insulin-sensitizing agents such as metformin may have independent effects in decreasing PAI-1 levels in patients with type 2 diabetic (Ersoy et al., 2008). Besides, activation of renin-angiotensin-aldosterone system (RASS) has also been found to be involved in the regulation of PAI-1 levels, and ACEI, such as quinapril, ramipril, and perindopril, have shown the ability to reduce PAI-1 level in both healthy people and hypertensive patients (Brown et al., 1998; 2002a; 2002b; Erdem et al., 1999). Statins can inhibit the production of PAI-1 by regulating a variety of signaling pathways as well (Ma et al., 2002; Laumen et al., 2008; Dunoyer-Geindre et al., 2011; Ni et al., 2013). However, the role of these drugs in cancer remains unclear. Apart from these traditional drugs, there are many more novel drugs under development. Due to the crucial role of reactive center loop (RCL) in inhibitory mechanism of PAI-1, several synthetic peptides that mimic various parts of the RCL of PAI-1 were developed (Eitzman et al., 1995; Kvassman et al., 1995; Xue et al., 1998; D’Amico et al., 2012). In general, peptides mimicking the C-terminal part of the loop can accelerate the irreversible transition of PAI-1 to its latent form, while peptides mimicking the N-terminal part can induce it to be cleaved (D’Amico et al., 2012). In addition, several RNA aptamers designed to block the interaction of pai-1 with its partner have been developed as well, and have shown the ability to reduce cancer migration, invasion, and angiogenesis (Blake et al., 2009; Damare et al., 2014; Fortenberry et al., 2016). Another class of PAI-1 inhibitors is small molecules. These compounds work by binding a common binding pocket within the flexible joint region of PAI-1 or by interfering structural elements within that region through interactions at the surface of PAI-1, thereby inducing the substrate behavior of PAI-1 and its conversion to an inert form (Egelund et al., 2001; Fjellström et al., 2013; Lin et al., 2013; Sillen et al., 2021). There are also lots of antibody based PAI-1 inhibitors, including antibodies and antibody derivatives. Their target sits and mechanisms are more extensive and complex than drugs mentioned above (Sillen and Declerck, 2020). However, although these different types of novel PAI-1inhibitors have been shown to be efficient *in vivo* or *in vitro*, their role and safety in cancers remain unclear and require more research and clinical trials to understand them, but they still promise a bright future for cancer therapies based on *SERPINE1*

Admittedly, there are several limitations to our study. First, there are some contradictory findings in our study. For example, in the Cox regression analysis, the result based on RECA-EU project data from ICGC database indicated a protective role of *SERPINE1* in renal cell carcinoma, which is contrary to the results of other datasets. We speculate that this may be related to the heterogeneity of samples from different datasets, and the sample size of this dataset (*n* = 91) is smaller than that of TCGA database, but further studies and follow-up are still needed to verify it. In addition, the results of different cancers are not always consistent in pan-cancer analysis. Therefore, further research focused on the differences in *SERPINE1* roles among cancers is needed as well. Second, although we have identified the prognostic value and possible action mechanisms of *SERPINE1* in cancers through correlation analysis in the present study, direct evidence supporting these conclusions are required. Finally, our research is mainly based on public databases, which may have inevitably introduced systemic bias; further experimental verification is therefore needed.

## 5 Conclusion

In the present study, we conducted a comprehensive multi-omics analysis of *SERPINE1* in pan-cancer, revealing its prognostic value and potential action mechanisms in cancers. The aberrant expression of *SERPINE1* is common in cancers and is associated with patient prognosis, cancer immunity, immunotherapy response and drug sensitivities. *SERPINE1* may thus be a promising new target for cancer diagnosis and treatment.

## Data Availability

The datasets presented in this study can be found in online repositories. The names of the repository/repositories and accession number(s) can be found in the article/Supplementary Material.
