# Supplementary figures and images for: Identification and validation of SERPINE1 as a prognostic and immunological biomarker in pan-cancer and in ccRCC
Source: Front Pharmacol. 2023 Aug 23;14:1213891. doi: 10.3389/fphar.2023.1213891 (PMC10482042; doi:10.3389/fphar.2023.1213891)

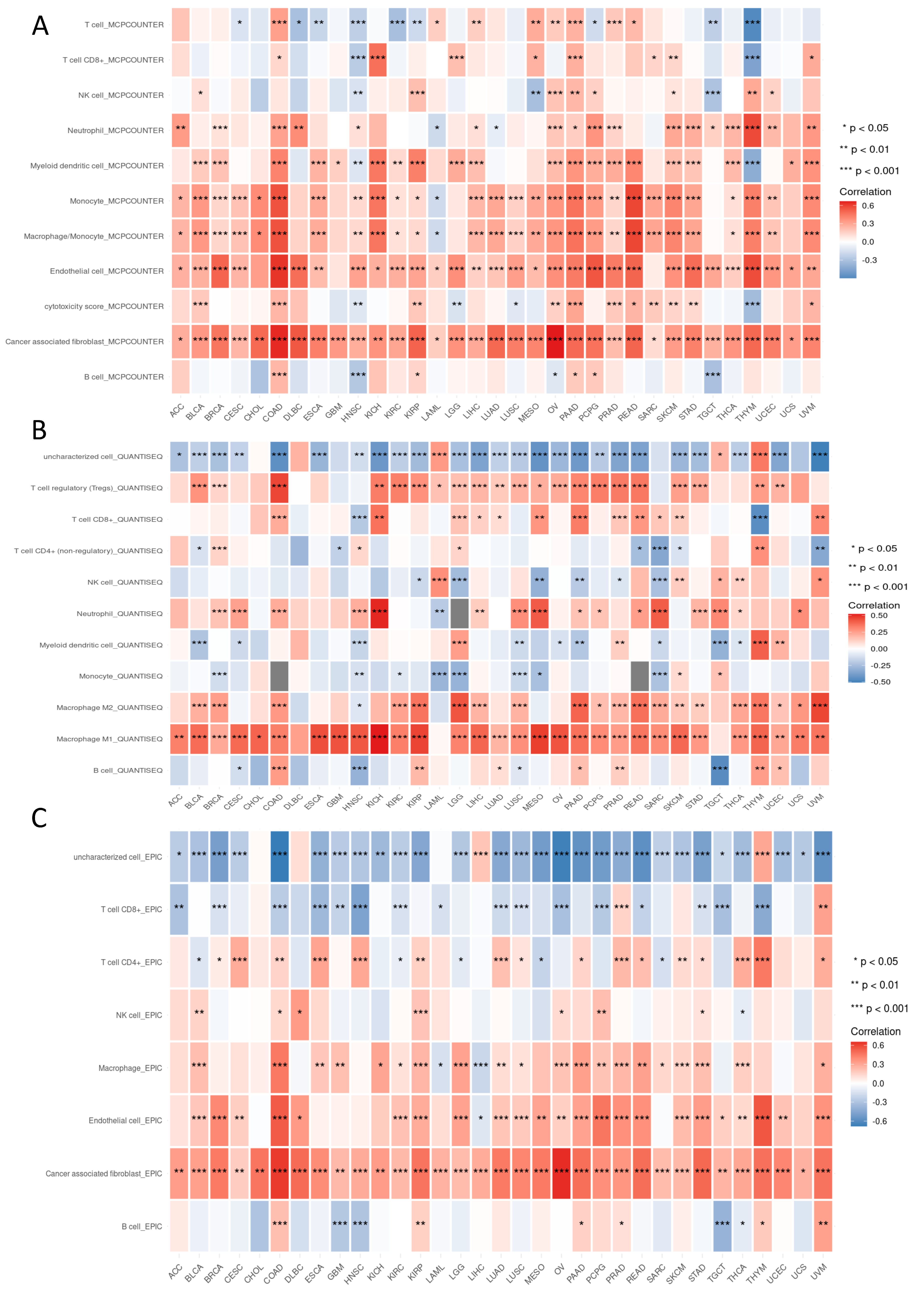

Supplement: Supplementary file 1 [file Image3.TIF]

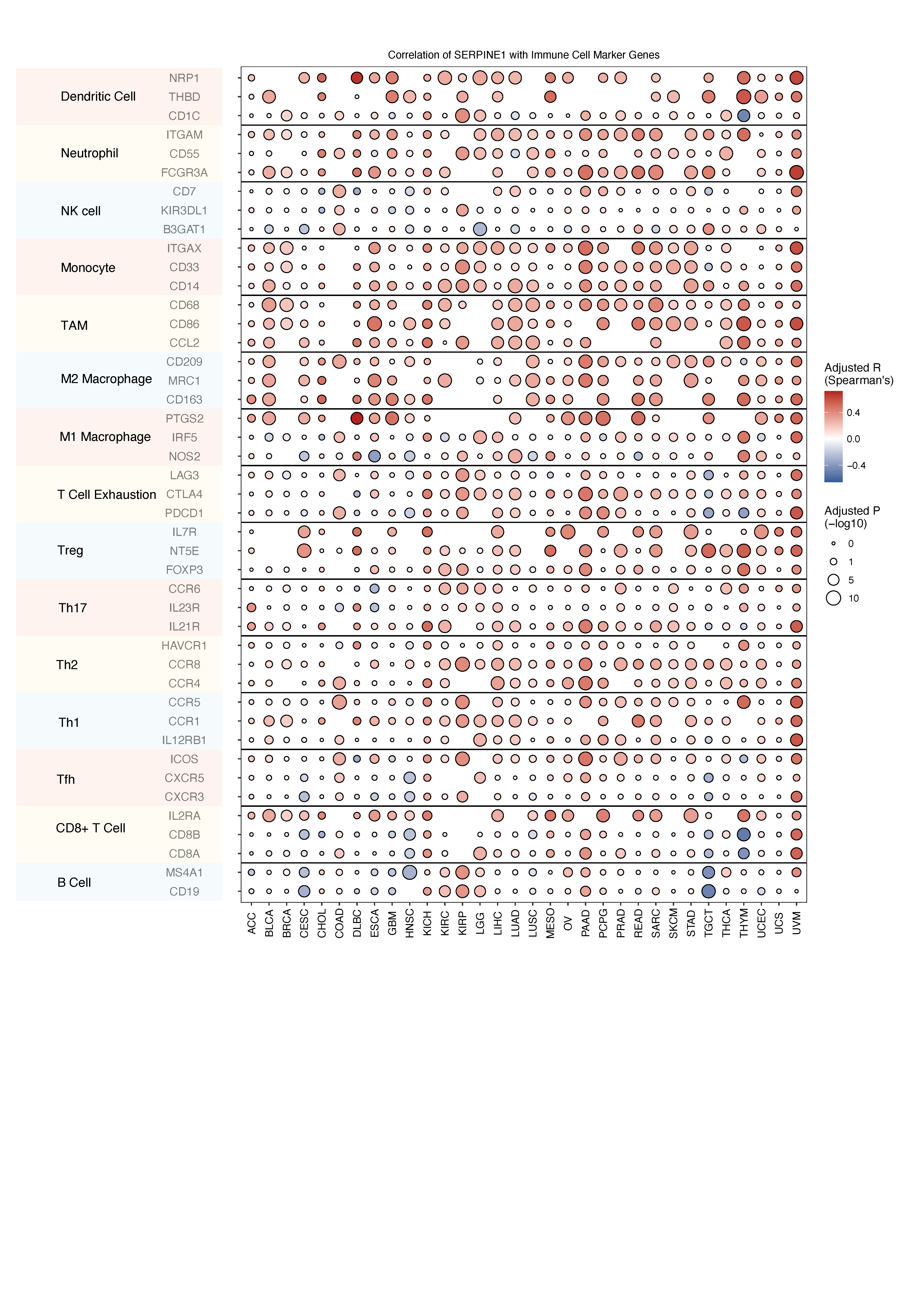

Supplement: Supplementary file 2 [file Image4.TIF]

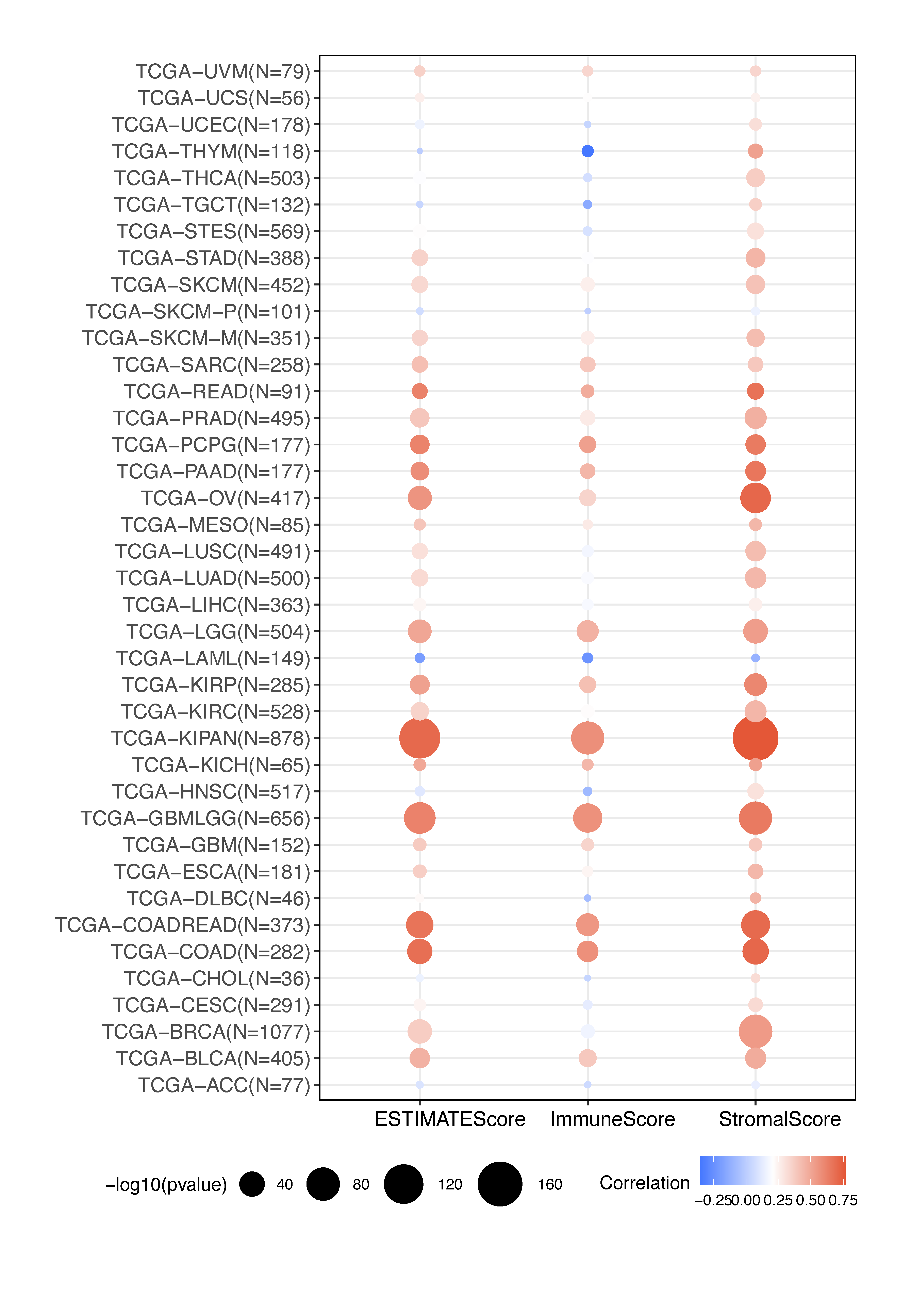

Supplement: Supplementary file 3 [file Image2.TIF]

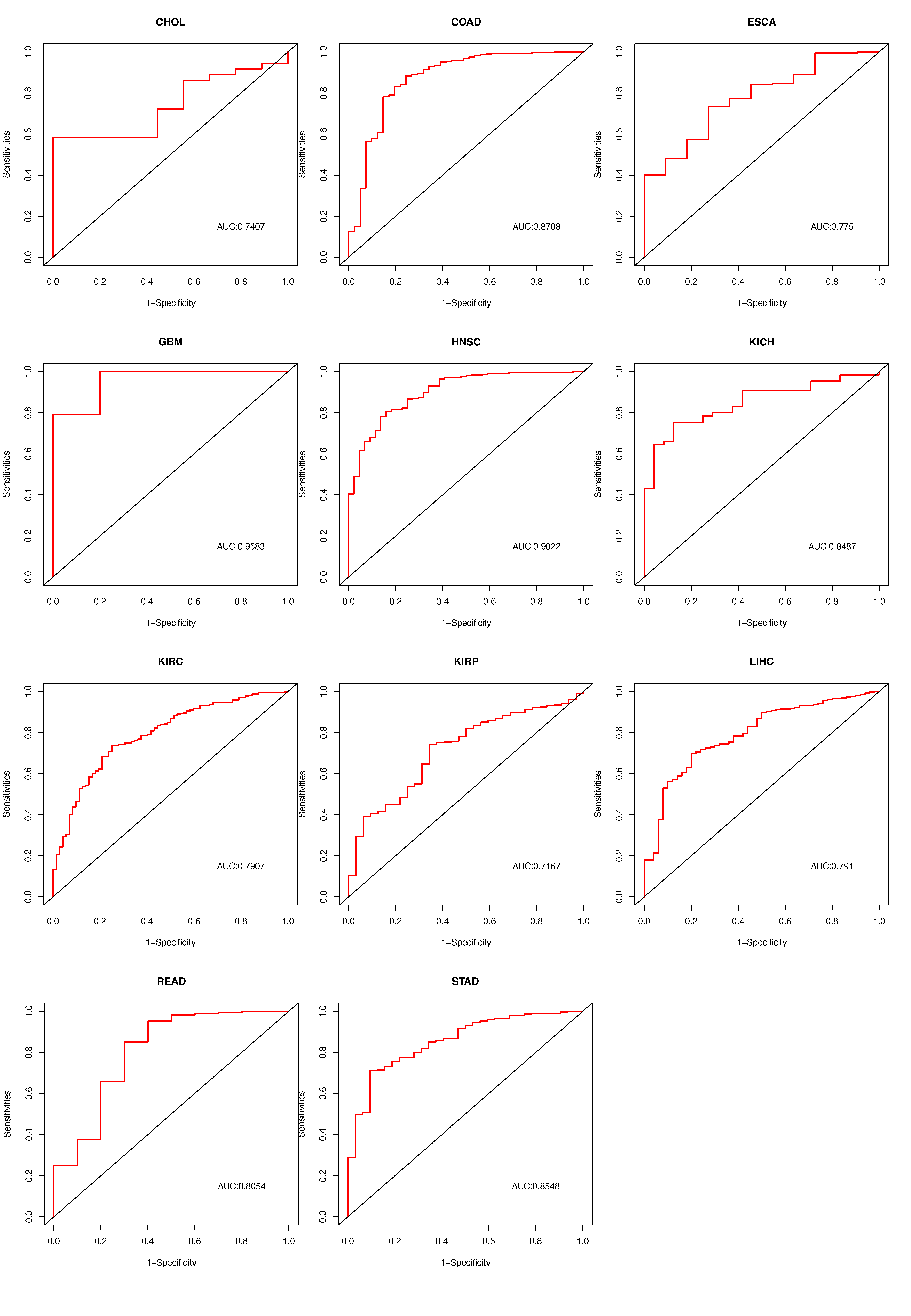

Supplement: Supplementary file 4 [file Image1.TIF]

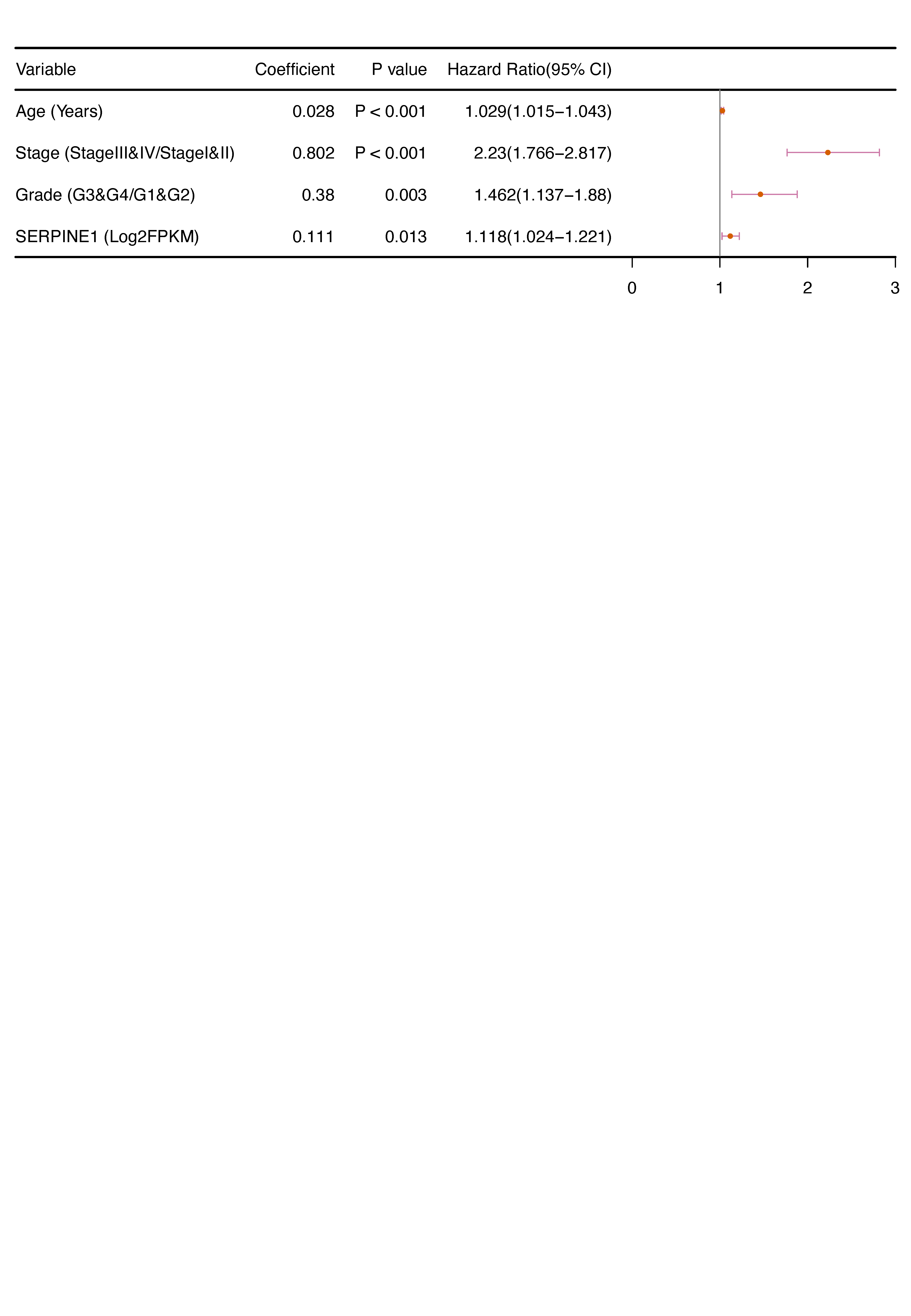

Supplement: Supplementary file 6 [file Image5.TIF]
